# The Impact of a Text Messaging Service (Tonsil-Text-To-Me) on Pediatric Perioperative Tonsillectomy Outcomes: Cohort Study With a Historical Control Group

**DOI:** 10.2196/39617

**Published:** 2022-09-20

**Authors:** Lori Wozney, Negar Vakili, Jill Chorney, Alexander Clark, Paul Hong

**Affiliations:** 1 Mental Health and Addictions Policy and Planning Nova Scotia Health Dartmouth, NS Canada; 2 Centre for Research in Family Health IWK Health Centre Halifax, NS Canada; 3 Mental Health and Addictions IWK Health Halifax, NS Canada; 4 Department of Psychiatry Dalhousie University Halifax, NS Canada; 5 Faculty of Medicine Dalhousie University Halifax, NS Canada; 6 Division of Otolaryngology IWK Health Halifax, NS Canada; 7 Division of Otolaryngology–Head & Neck Surgery Department of Surgery Dalhousie University Halifax, NS Canada

**Keywords:** tonsillectomy, otorhinolaryngology, text messaging, caregivers, surgery, perioperative, patient discharge, aftercare, short messaging service, pain management, mobile phone

## Abstract

**Background:**

Tonsillectomy is a common pediatric surgical procedure performed in North America. Caregivers experience complex challenges in preparing for their child’s surgery and coordinating care at home and, consequently, could benefit from access to educational resources. A previous feasibility study of *Tonsil-Text-To-Me*, an automated SMS text messaging service that sends 15 time-sensitive activity reminders, links to nutrition and hydration tips, pain management strategies, and guidance on monitoring for complications, showed promising results, with high levels of caregiver satisfaction and engagement.

**Objective:**

This study aimed to pilot-test *Tonsil-Text-To-Me* in a real-world context to determine whether and how it might improve perioperative experiences and outcomes for caregivers and patients.

**Methods:**

Caregivers of children aged 3 to 14 years undergoing tonsillectomy were included. Data from a historical control group and an intervention group with the same study parameters (eg, eligibility criteria and surgery team) were compared. Measures included the Parenting Self-Agency Measure, General Health Questionnaire-12, Parents’ Postoperative Pain Measure, Client Satisfaction Questionnaire-8, and engagement analytics, as well as analgesic consumption, pain, child activity level, and health service use. Data were collected on the day before surgery, 3 days after surgery, and 14 days after surgery. Participants in the intervention group received texts starting 2 weeks before surgery up to the eighth day after surgery. Descriptive and inferential statistics were used.

**Results:**

In total, 51 caregivers (n=32, 63% control; n=19, 37% intervention) who were predominately women (49/51, 96%), White (48/51, 94%), and employed (42/51, 82%) participated. Intervention group caregivers had a statistically significant positive difference in Parenting Self-Agency Measure scores (*P*=.001). The mean postoperative pain scores were higher for the control group (mean 10.0, SD 3.1) than for the intervention group (mean 8.5, SD 3.7), both of which were still above the 6/15 threshold for clinically significant pain; however, the difference was not statistically significant (*t*_39_=1.446; *P*=.16). Other positive but nonsignificant trends for the intervention group compared with the control group were observed for the highest level of pain (*t*_39_=0.882; *P*=.38), emergency department visits (*χ*^2^_2_=1.3; *P*=.52; Cramer V=0.19), and other measures. Engagement with resources linked in the texts was moderate, with all but 1 being clicked on for viewing at least once by 79% (15/19) of the participants. Participants rated the intervention as highly satisfactory across all 8 dimensions of the Client Satisfaction Questionnaire (mean 29.4, SD 3.2; out of a possible value of 32.0).

**Conclusions:**

This cohort study with a historical control group found that *Tonsil-Text-To-Me* had a positive impact on caregivers’ perioperative care experience. The small sample size and unclear impacts of COVID-19 on the study design should be considered when interpreting the results. Controlled trials with larger sample sizes for evaluating SMS text messaging interventions aimed to support caregivers of children undergoing tonsillectomy surgery are warranted.

## Introduction

Tonsillectomy is one of the most common pediatric surgical procedures performed in North America, comprising 16% of all ambulatory surgeries performed on the pediatric population [1]. As the surgery is frequently performed on an outpatient basis, most of the perioperative care is undertaken by caregivers at home [2]. Caregivers can become confused, anxious, or overwhelmed because of a lack of knowledge about how to prepare for their child’s surgery; how to monitor for complications such as postoperative pain, nausea, or reduced oral intake; and how to administer appropriate pain medication [3,4]. These uncertainties can contribute to the 33% of caregivers who make unscheduled health care visits to the clinic or emergency department (ED) after surgery [5]. In a study evaluating >36,000 tonsillectomies with or without adenoidectomies, 7% of patients revisited the hospital, and 1% of patients revisited a second time. Acute pain accounted for 18% of the first revisits and 11% of the second revisits, whereas fever and vomiting or dehydration were the primary diagnoses in 28% and 18% of the revisits, respectively [6]. A large proportion of return visits to hospitals are treat-and-release visits that may have been avoided through more adequate symptom control at home [7].

Efforts to support families through this perioperative period typically include health care providers offering verbal instructions or sharing web-based and printed resources and pamphlets. Studies have shown that caregivers typically correctly recall only parts of the information explained to them at the clinic [8], and almost half of this information is remembered incorrectly [9,10]. With >90% of adults in North America owning internet-enabled devices, it is common for caregivers to use the internet to learn about their child’s health issues or seek alternative treatment options [11,12]. However, the reliability, quality, and readability of the evidence found in these web-based resources, particularly for tonsillectomy, may be questionable or difficult to understand [13-15]. By following outdated or inaccurate information, caregivers risk making decisions that can negatively affect recovery, such as underdosing their child’s postoperative analgesics [16]. Improving timely access to quality perioperative education might help to better prepare families and reduce these potential negative effects [17].

SMS text messages are convenient, cost-effective, asynchronous (ie, can be read by participants at times they prefer), and do not require labor-intensive face-to-face contact. SMS text messaging interventions have been shown to improve not only medical appointment adherence but also treatment compliance for a range of clinical contexts [18,19]. Leveraging clinical recommendations from our previous Delphi study [20] and results of the early feasibility study [21], our team developed an automated SMS text messaging service, *Tonsil-Text-To-Me* (TTTM), to provide just-in-time support to caregivers across the perioperative pathway. The results of the feasibility and usability study showed that caregivers viewed the TTTM system as an improvement over the standard model of information delivery with no safety or security concerns, and although the SMS text messages were fully automated, participants saw them as reinforcing a sense of support from their health care team.

The objectives of this study were to investigate whether TTTM was effective at decreasing caregivers’ level of preoperative anxiety and distress, reducing postsurgery health care use, improving pain management, and having a positive impact on child outcomes (eg, hydration, level of activity, and pain-related behavior). We expected that caregivers receiving TTTM would report high satisfaction levels consistent with the feasibility study results.

## Methods

### Study Design

After receiving institutional review board approval, we conducted a prospective quasi-experimental pilot study to compare data from a historical usual care group (control) with a group receiving TTTM (intervention) in addition to usual care. Although not involving random allocation, the historical control group data offer a useful comparator for early pilot studies where researchers are interested in refining parameter estimates for larger controlled trials [21]. The original study plan aligned with criteria for when a historical control group would have less risk to validity (eg, precisely defined standard treatment, same participant eligibility for both groups, same methods of evaluation, and performed in the same organization) [22,23]. As this was an exploratory study with limited funding, we set a sample size goal based on guidelines [24] for 30 participants in each group. Data collection occurred at time point 1 (T1; the day before the surgery), time point 2 (T2; 3 days after surgery), and time point 3 (T3; 14 days after surgery).

### Setting and Population

The study took place at a pediatric otolaryngology clinic within a teaching hospital in Nova Scotia, Canada (IWK Health Centre). More than 300 tonsillectomies were performed at this clinic in 2017, the year preceding this study. Surgeries were often scheduled 3 to 6 months after the consultation visit, resulting in a large time gap in which usual care instruction booklets could be misplaced or critical information forgotten. Caregivers of children aged 3 to 14 years who received a surgical referral at the IWK Health Centre for tonsillectomy with or without adenoidectomy were approached. Caregivers aged ≥18 years, with a cell phone, and who were able to understand the SMS text messages in English were eligible. We excluded families from the study if the child had complex medical needs beyond routine tonsillectomy surgical care; a peritonsillar abscess or suspicion of malignancy; nonelective indications; and complex chronic conditions, craniofacial abnormalities, diabetes, or a disorder in hemostasis. The inclusion criteria for the control and intervention groups were the same. Informed consent was obtained from all participants.

### Intervention

The TTTM service sent 15 texts to caregivers over a 3-week period, including 8 before surgery, 1 on the day of surgery, and 6 during the week after surgery (Textbox 1). The automated service sent messages timed to the surgery date so that time-sensitive information (eg, what to bring to the hospital on the day of surgery) arrived at the right time (eg, the evening before surgery). The message content was based on evidence-based recommendations [15] and included reminders on when to start or stop activities, tips on pain management, and recommendations on when to follow up with a provider. To support active engagement with the content of the brief messages (122-135 characters), 8 texts also included a link to an external resource (eg, web-based tour of the day surgery unit, map of directions to the hospital, and a list of soft food). Of the 15 messages, 10 (67%) were set to be delivered in the morning, and 5 (33%) were set to be delivered in the evening.

**Tonsil Text-to-Me SMS text messaging and data collection schedule. ENT: ear, nose, and throat.**

**Before surgery**

- 14 days before: Acknowledge sign-up, clinic contact number, and how to stop receiving texts
- 10 days before: Link to a coloring book story about day surgery for the child
- 7 days before: Information on stopping medication
- 6 days before: Link to the day surgery web-based tour video
- 4 days before: Link to the checklist for what to bring to the hospital
- 3 days before: Link to a list of soft food ideas
- 2 days before: Link to pain management tips, how to cancel surgery, and reminder that it is okay for the child to eat as usual that day
- 1 day before: Reminder on when to stop solid foods and link to parking instructions for hospital

**Time point 1 data collection (day before surgery)**

**Day of surgery**

- Link to checklist for what to bring to the hospital and tips on how to ask their child about their pain

**After surgery**

- 1 day after: Link to tips on encouraging food and fluid intake and clinic contact number
- 2 days after: Information on physical symptoms typical of peak pain period and guidance on resumption of physical activity
- 3 days after: Information on typical peak pain, pain occurrences, and tips on pain management

**Time point 2 data collection (3 days after surgery)**

- 5 days after: Information on when the child might return to school
- 7 days after: Information on resuming physical activities
- 8 days after: Provided information on the ENT Clinic helpline in case of continued pain and discomfort.

**Time point 3 data collection (14 days after surgery)**

### Measures

#### Demographics

Several demographic measures were collected at baseline: age, gender, ethnicity, employment status, education level, current use of technology, and preferences for using technology in different health-related capacities.

#### Caregiver Self-efficacy

Preoperative caregiver self-efficacy was measured at T1 using 3 problem-solving items from the Parenting Self-Agency Measure (PSAM) [25] (ie, “I feel sure of myself as a parent,” “I can solve most problems between my child and me,” and “when things are going badly between my child and me, I keep trying until things begin to change”). The PSAM is a self-report measure of general self-efficacy for parents of children aged 3 to 12 years. Respondents rated each of the 3 items using a 5-point Likert scale ranging from 1=never to 5=always. A total score between 3 and 15 was computed.

#### Caregiver Distress

Preoperative caregiver distress was measured at T1 using the well-validated short form of the General Health Questionnaire-12 (GHQ-12) [26]. The GHQ-12 covers several domains associated with a person’s level of distress and is worded in such a way as to comprise 6 positive and 6 negative items. Response items are scored on a 4-point scale (ranging from 0 to 3), and a global score between 0 and 36 is calculated, with higher scores indicating higher levels of distress.

#### Child’s Pain

At T2 and T3, caregivers were asked to report their child’s average level of pain in the past 24 hours and the highest level of pain in the past 24 hours on a scale of 0 (no pain) to 10 (worst pain). The well-established 15-item Parents’ Postoperative Pain Measure (PPPM) [27] was used to measure caregiver-reported pain-related behavior of their child at T2. A sum score was computed by tallying the number of *yes*=1 and *no*=0 responses for a total score of 15. As per guidelines, a score of 6/15 signified clinically significant pain [28].

#### Child’s Activity

As a proxy measure of fluid intake, we asked caregivers at T2 to report “yes” or “no” as to whether their child had urinated at least twice in the previous 24-hour period. The child’s activity level was measured at T2 and T3 by asking caregivers to report the level of physical activity on a 4-item scale (ie, bedridden, sluggish but walking, easily tired but active, or normal) during the past 24 hours.

#### Analgesic Therapy

Caregivers reported the number of doses per type of analgesic (eg, acetaminophen, ibuprofen, and morphine) administered within the past 24-hour period at T2 and T3.

#### Health Care Use

At T3, caregivers were asked to report on the number of postoperative ED visits; hospitalizations; family physician visits; calls to ear, nose, and throat (ENT) nurses or surgeons; acute or unplanned clinic visits; calls to 811 (local nonurgent health care advice line); and the number of antibiotic courses prescribed in relation to the tonsillectomy since surgery day.

#### Satisfaction and Intervention Engagement

Intervention group participants were asked to rate their satisfaction with the TTTM service at T3 using the Client Satisfaction Questionnaire [29], which is a unidimensional, 8-item measure used worldwide to assess client or patient satisfaction with health services. Responses are scored from 1 to 4, and thus, the possible total scores ranged from 8 to 32. Higher scores indicate greater satisfaction. Engagement with the TTTM messages was operationalized as the number of texts received, number of clicks on embedded links, and number of caregivers who opted out of the service by texting “STOP” before all texts were received. Aggregate engagement analytics were compiled at T3 through the SMS text messaging platform.

### Recruitment and Enrollment

The original study plan was to begin recruitment for the intervention group immediately after data collection for the historical control group. However, institutional IT approval and the privacy process related to technical infrastructure caused significant delays, further compounded by the COVID-19 pandemic’s impacts on clinical research [30].

Control group cohort data was collected over a 10-month period starting in 2017. A 4-month period was used for active recruitment, there was a 3- to 4-month wait for surgery, and the postsurgery follow-up period lasted for ≥2 weeks. Control group participants (ie, caregivers) were recruited through advertisements displayed at the clinic and through clinic nurses who introduced the study to caregivers. In addition, caregivers were able to self-enroll by visiting our web-based recruitment site and completing a 5-minute guided screening and web-based consent process. Once enrollment was confirmed, the research coordinator generated a study ID in REDCap (Research Electronic Data Capture; Vanderbilt University) [31], and an automated questionnaire schedule sent surveys to caregivers on the day before the surgery (T1), during the peak pain period on day 3 (T2), and 14 days after surgery (T3). REDCap also sent 2 reminder emails for surveys that were not completed.

Intervention group data collection ran from May 2021 to December 2021. Recruitment flow was adjusted for the intervention group to allow for flexibility in changing COVID-19 pandemic precautions and hospital restrictions; for example, as in-clinic recruitment was not possible, distance-delivered recruitment materials were developed. Potential participants were identified by screening the surgical wait-list for families whose surgery dates fell within the study timeline. A postcard with study details was mailed, and a follow-up phone call was made. After informed consent was confirmed, the research coordinator generated a study ID in REDCap, and an automated questionnaire schedule sent surveys to caregivers on the day before the surgery (T1), during the peak pain period on day 3 (T2), and 14 days after surgery (T3). A booking clerk entered the participant’s information into the surgery booking interface, where they flagged the study participant to receive the texts. Using a secure file transfer protocol, we sent a daily report for those enrolled in the TTTM intervention to the SMS text messaging service vendor SimplyCast. SimplyCast’s secure SMS text messaging service sent SMS text messages with periodic embedded links per the defined schedule to caregivers based on surgery data outlined in the SMS text message schedule (see the *Results* section).

### Statistical Analysis

We used SPSS software (IBM Corp) [32] and Jeffreys’s Amazing Statistics Program [33] for data analysis. Standard descriptive statistics, including means, SDs, frequencies, and percentages, were used to summarize the continuous preoperative and postoperative measures as appropriate. Differences between the 2 groups were tested with paired-sample *t* tests (2-tailed) or chi-square tests where appropriate. Where assumptions of normal distribution and equality of variance were violated, Mann-Whitney *U* tests were used. Effect sizes were extracted (ie, Cohen *d*, Cramer V, odds ratios [ORs], and rank-biserial correlation) where applicable. All statistical tests were performed using 2-tailed tests at the 0.05 level of significance. Analysts were not blinded to group allocation.

### Ethics Approval

This study has been funded by an IWK Health Centre Translating Research Into Care grant and has been approved by the IWK Health Centre Research Ethics Board (1021845).

## Results

### Overview

**Figure 1:**
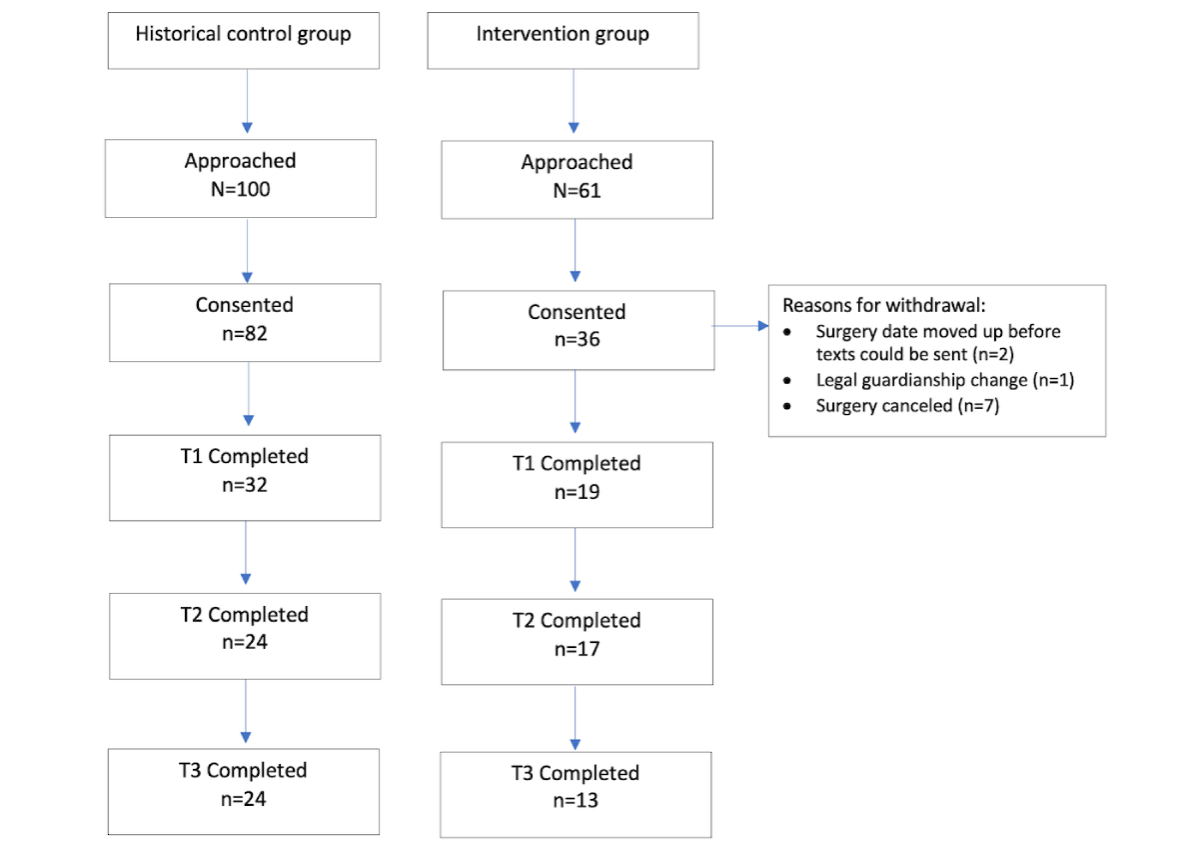
Flowchart of study participants in the historical control and intervention groups.

An overview of recruitment and enrollment is presented in the Figure 1 flow diagram. A total of 100 caregivers were approached during control group data collection and 61 during intervention group data collection. Approximately 82% (82/100) consented to participate in the historical control group, and 59% (36/61) consented to participate in the intervention group. Approximately 28% (10/36) of intervention group participants withdrew before T1 data collection for reasons that included changed or canceled surgery dates and changes in legal guardianship status.

### Demographics

An overview of baseline demographics is presented in Table 1. All but 1 participant were women caregivers. Most were White, employed with a university degree, and living in a household with ≥2 children. There were no significant group differences at baseline regarding the age of the caregiver (*χ^2^*_3_=3.3; *P*=.35), gender (*χ^2^*_2_=3.5; *P*=.17), education level (*χ^2^*_3_=5.8; *P*=.12), ethnicity (*χ^2^*_2_=1.9; *P*=.39), employment status (*χ^2^*_2_=3.0; *P*=.28), or number of children in the household (*χ^2^*_2_=1.0; *P*=.60).

Preferences for using SMS text messages for different health care service use contexts are reported in Table 2. Respondents in both groups reported high use of SMS text messaging in daily life, with 98% (50/51) reporting that they send SMS text messages at least once a day. When asked to rank the top 3 reasons for using their mobile phones, respondents in both control and intervention groups indicated that receiving and sending SMS text messages was the number 1 reason (32/32, 100%, and 19/19, 100%, respectively), followed by receiving and making phone calls (22/32, 69%, and 17/19, 90%, respectively). Being able to receive appointment reminders (49/51, 96%) and consult with health care professionals (36/51, 71%) were among the top ways that respondents wanted to use their mobile phones.

**Table 1:** Baseline demographic characteristics of caregivers (N=51).

Characteristics		Control group (n=32), n (%)	Intervention group (n=19), n (%)
**Age (years)**			
	18 to 25	0 (0)	1 (5)
	26 to 35	11 (34)	9 (47)
	36 to 45	20 (63)	9 (47)
	≥46	1 (3)	0 (0)
**Gender**			
	Woman	32 (100)	17 (90)
	Man	0 (0)	1 (5)
	Other or prefer not to say	0 (0)	1 (5)
**Ethnicity**			
	White	31 (97)	17 (90)
	Middle Eastern	1 (3)	1 (5)
	African Canadian, African American, or Caribbean	0 (0)	1 (5)
**Highest educational level**			
	High school or less	3 (9)	4 (21)
	College diploma	10 (31)	2 (10)
	University degree	18 (56)	10 (53)
	Other	1 (3)	3 (16)
**Employment**			
	Unemployed	3 (9)	5 (26)
	Employed	28 (87)	14 (74)
	Prefer not to say	1 (3)	0 (0)
**Number of children in the household**			
	1	7 (22)	5 (26)
	2	18 (56)	8 (42)
	≥3	7 (22)	6 (32)

**Table 2:** Baseline technology use and preferences of caregivers (N=51).

Technology uses and preferences			Control group (n=32), n (%)	Intervention group (n=19), n (%)
**Number of texts sent per week**				
	At least once a day		32 (100)	18 (95)
	More than once a week but less than once a day		0 (0)	1 (5)
	Less than once per week		0 (0)	0 (0)
**Would you like to use your mobile phone for the following**				
	**Receive appointment and vaccination reminders**			
		Yes	30 (94)	19 (100)
		No	2 (6)	0 (0)
	**Consult with physicians and nurses**			
		Yes	23 (72)	13 (68)
		No	9 (28)	6 (32)
	**Get help sticking with a medication regimen**			
		Yes	12 (37)	4 (21)
		No	20 (63)	15 (79)
	**Receive test results**			
		Yes	23 (72)	13 (68)
		No	9 (28)	6 (32)
	**Talk with a professional about health concerns**			
		Yes	16 (50)	9 (47)
		No	16 (50)	10 (53)
	**Access emergency services**			
		Yes	20 (63)	7 (37)
		No	12 (37)	12 (63)

### Caregiver Self-efficacy and Distress

Out of a possible total score of 15, at T1, the mean scores on the 3 PSAM items were 12.5 (SD 1.1) for the control group and 13.7 (SD 1.1) for the intervention group. A Mann-Whitney *U* test indicated that the mean scores on parenting self-efficacy were significantly higher for the intervention group, with a small effect size (*U*=136.50; *P*=.002; *r*_rb_=0.53, 95% CI –0.73 to –0.24). Overall, on the GHQ-12, both control (mean 2.53, SD 0.57) and intervention group (mean 2.42, SD 0.61) participants reported challenges in feeling “capable of making decisions” and in feeling that they were “playing a useful part in things” (Table 3). The effect size for mean differences on the GHQ-12 in this analysis was small (Cohen *d*=0.32, 95% CI –0.26 to 0.88), and the independent-sample *t* test indicated a nonsignificant difference (*t*_49_=1.090; *P*=.28).

**Table 3:** Caregivers’ GHQ-12^a^ scores at time point 1 (N=51).

GHQ-12 items (have you done the following)	Control group (n=32), mean (SD)^a^	Intervention group (n=19), mean (SD)^a^
Been able to concentrate on what you were doing	2.09 (0.86)	2.16 (0.83)
Lost much sleep over worry	1.16 (1.02)	1.84 (0.96)
Felt that you are playing a useful part in things	2.31 (0.69)	2.32 (0.67)
Felt capable of making decisions about things	2.53 (0.57)	2.42 (0.61)
Felt constantly under strain	1.37 (0.91)	1.32 (1.20)
Felt you could not overcome your difficulties	0.72 (0.77)	1.05 (1.08)
Been able to enjoy your normal day-to-day activities	2.16 (0.57)	2.16 (0.83)
Been able to face your problems	2.25 (0.62)	1.06 (0.80)
Been feeling unhappy or depressed	1.06 (0.80)	1.21 (1.08)
Been losing confidence in yourself	0.72 (0.73)	0.84 (1.02)
Been thinking of yourself as worthless	0.31 (0.54)	0.84 (1.02)
Been feeling reasonably happy	2.09 (0.69)	1.74 (0.93)
Overall score	18.78 (3.02)	20.37 (3.34)

^a^GHQ-12: General Health Questionnaire-12.

### Child’s Pain

At T2, on a scale of 0 (no pain) to 10 (worst pain), the control group reported a slightly lower average level of pain (mean 4.38, SD 1.76) than the intervention group (mean 4.65, SD 2.26). The mean score for the highest level of pain at T2 was 7.37 (SD 1.88) for the control group and slightly lower at 6.70 (SD 2.97) for the intervention group. Independent-sample *t* tests did not indicate a significant difference between the groups, and only small effects were observed on the average level of pain (*t*_39_=–0.433; *P*=.67; Cohen *d*=0.14, 95% CI –0.76 to 0.49) and the highest level of pain (*t*_39_=0.882; *P*=.38; Cohen *d*=0.28, 95% CI –0.34 to 0.90).

The most frequently reported pain-related change in behavior at T2 in the control group was eating less than usual (22/24, 92%). In the intervention group, the most common behavior change was wanting to be close to their caregiver more than usual (14/17, 82%) and eating less (14/17, 82%; Table 4). The least frequently reported pain-related change in behavior for the control group was acting more worried than usual (8/24, 33%), and for the intervention group, it was the child taking medication when they normally refuse (3/17, 18%). The mean PPPM score was higher for the control group (mean 10.0, SD 3.1) than for the intervention group (mean 8.5, SD 3.7), both of which were still above the 6/15 threshold for clinically significant pain. An independent-sample *t* test did not report a significant difference in PPPM scores (*t*_39_=1.446; *P*=.16), although a small effect size was found (Cohen *d*=0.46, 95% CI –0.02 to 1.08).

**Table 4:** Frequency of caregivers’ endorsement of PPPM^a^ items at time point 2 (N=41).

PPPM items (when your child was recovering from surgery, did she or he do the following?)	Control group (n=24), n (%)	Intervention group (n=17), n (%)
Whine or complain more than usual	17 (71)	10 (59)
Cry more easily than usual	15 (63)	9 (53)
Play less than usual	21 (88)	12 (71)
Not do the things she or he normally does	15 (63)	12 (71)
Act more worried than usual	8 (33)	6 (35)
Act more quiet than usual	17 (71)	11 (65)
Have less energy than usual	18 (75)	11 (65)
Refuse to eat	12 (50)	10 (59)
Eat less than usual	22 (92)	14 (82)
Hold the sore part of his or her body	13 (54)	7 (41)
Try not to bump the sore part of his or her body	15 (63)	6 (35)
Groan or moan more than usual	16 (67)	8 (47)
Look more flushed than usual	16 (67)	11 (65)
Want to be close to you more than usual	21 (88)	14 (82)
Take medication when she or he normally refuses	14 (58)	3 (18)

^a^PPPM: Parents’ Postoperative Pain Measure.

### Analgesic Therapy

Analgesic therapy was consistent across the groups. At T2, caregivers in both the control and intervention groups reported administering on average 3.75 (SD 0.61) and 3.59 (SD 1.73) doses of acetaminophen, respectively, and 3.46 (SD 1.06) and 3.59 (SD 1.73) doses of ibuprofen, respectively, within the previous 24 hours (range 0-8; Table 5). Across both groups at T3 (14 days after surgery), only one of the caregivers reported offering analgesics within the previous 24-hour period. Chi-square group difference tests on use or nonuse of medication did not indicate a significant association, although small effects were demonstrated at both T2 (*χ^2^*_1_=0.9; *P*=.32; OR 0.33, 95% CI 0.01-8.79) and T3 (*χ^2^*_1_=0.8; *P*=.36; OR 0.39, 95% CI 0.01-10.37).

**Table 5:** Average analgesic doses administered in the previous 24 hours (T2^a^ and T3^b^; N=76).

Dosages		Control group	Intervention group
**T2 (3 days after surgery)^c^**			
	Acetaminophen, mean (SD; range)	3.75 (0.61; 2-4)	3.59 (1.73; 0-8)
	Ibuprofen, mean (SD; range)	3.46 (1.06; 0-4)	3.59 (1.73; 0-8)
	Morphine, mean (SD; range)	1.12 (1.15; 0-4)	0.59 (1.06; 0-4)
**T3 (14 days after surgery)^d^**			
	Acetaminophen, mean (SD; range)	0.05 (0.21; 0-1)	0 (0; 0)
	Ibuprofen, mean (SD; range)	0.05 (0.21; 0-1)	0 (0; 0)
	Morphine, mean (SD; range)	0 (0; 0)	0 (0; 0)

^a^T2: time point 2.

^b^T3: time point 3.

^c^Control group: n=24; intervention group: n=17.

^d^Control group: n=22; intervention group: n=13.

### Child’s Activity

In terms of fluid intake, all caregivers reported that their children had urinated at least twice in the past 24 hours. In addition, at T2, caregivers in the control group reported that 13% (3/24) of the children were at their normal level of activity in the past 24 hours compared with 24% (4/17) in the intervention group. A caregiver in each group reported that their child was bedridden. Most caregivers in the control group reported that their child was “easily tired but active” (16/24, 67%), whereas caregivers in the intervention group reported that their child was “sluggish but walking” (6/17, 35%) or “easily tired but active” (6/17, 35%). By T3, most (21/23, 91% control group; 13/13, 100% intervention group) of the caregivers reported that their children had returned to normal activity levels. We created a dichotomous variable of normal activity versus reduced activity (ie, easily tired, sluggish, or bedridden). The chi-square group difference for normal activity and reduced activity showed no significant differences at T2 (*χ^2^*_1_=0.8; *P*=.35; OR 2.15, 95% CI 0.41-11.20).

### Health Service Use

Hospital admissions were reported by 13% (3/23) of the respondents in the control group and 8% (1/13) of those in the intervention group, with visits to the ED reported by 17% (4/23) and 8% (1/13), respectively. The number of calls to the ENT clinic, family physicians, or 811 (local health information phoneline) was higher in the control group (8/23, 35%) than in the intervention group (4/13, 31%). Antibiotic prescriptions were reported by 9% (2/23) of the caregivers in the control group and 15% (2/13) of the caregivers in the intervention group. However, chi-square and Cramer V tests showed no significant differences and only small associations for hospital admissions lasting for <24 hours (*χ^2^*_1_=0.01; *P*=.92; Cramer V=0.02), lasting for >24 hours (*χ^2^*_1_=0.6; *P*=.45; Cramer V=0.13), ED visits (*χ*^2^_2_=1.3; *P*=.52; Cramer V=0.19), visits to outpatient walk-in clinics (*χ^2^*_1_=1.2; *P*=.27; Cramer V=0.18), calls to the ENT clinic (*χ^2^*_2_=2.1; *P*=.35; Cramer V=0.24), or calls to 811 or family physician (*χ^2^*_1_=1.85; *P*=.17; Cramer V=0.23).

### Satisfaction and Engagement

The results of the Client Satisfaction Questionnaire-8 showed high levels of satisfaction with TTTM across all 8 dimensions (Table 6). The mean total satisfaction score, out of a possible 32, was 29.4 (SD 3.6, range 24.0-32.0).

All caregivers engaged with the full TTTM intervention, and none texted “STOP” to cease the messages. Engagement with the linked resources within the texts was moderate, with 90% (9/10) of the embedded links within the texts being viewed at least once by 79% (15/19) of the participants. All participants (19/19, 100%) viewed the web-based tour video and both checklists of what to bring to the hospital. Approximately 79% (15/19) viewed the presurgery tips on nonpharmacological postsurgery pain management; however, only 58% (11/19) viewed the postsurgery link regarding how to ask their child about their level of pain (Table 7).

**Table 6:** Results of the CSQ-8^a^ (N=13).

CSQ-8 dimensions	Values, mean^b^ (SD)
Quality of service	3.62 (0.51)
Kind of service you wanted	3.69 (0.48)
The extent to which the program met your needs	3.69 (0.48)
Recommend the program to a friend	3.69 (0.48)
Satisfaction with the amount of help received	3.77 (0.44)
Services helped you to deal with problems	3.54 (0.52)
Overall satisfaction with the service	3.69 (0.48)
Return to the program for help	3.69 (0.48)

^a^CSQ-8: Client Satisfaction Questionnaire-8.

^b^Highest possible score=4.

**Table 7:** Participants’ engagement with the linked resources within the SMS text messages.

Embedded links topic	Intervention group (n=19), n (%)
Coloring book	11 (58)
Web-based tour	19 (100)
Checklist	19 (100)
Soft food list	15 (79)
Postsurgery pain	15 (79)
Parking	13 (69)
Eating and drinking	9 (48)
Asking about pain	11 (58)

## Discussion

### Principal Findings

In this study, a brief 15-message TTTM intervention that was delivered adjunct to usual care during the COVID-19 pandemic revealed high uptake and engagement. A positive, significant difference in preoperative caregiver self-efficacy was found, suggesting that SMS text messages may have helped caregivers to develop positive expectations regarding their ability to handle postoperative activities with their child. Furthermore, caregivers receiving the texts reported improvements over usual care related to the highest level of child’s pain intensity, child’s pain-related behavior, health care use, and child’s return to normal activity levels, although statistical significance was not noted. These results are not unlike other SMS text messaging intervention studies that target the perioperative experiences of adults [34,35] and suggest that pediatric perioperative pathways are a rich area for further research. In the following sections, we detail the strengths and limitations of this study, as well as future lines of inquiry.

The study has several strengths. First, research on the use of technology to support perioperative education for pediatric tonsillectomy is nascent, despite being one of the most frequently performed pediatric surgeries. A systematic review [36] of phone- and internet-based pain and recovery support programs for pediatric tonsillectomy found only 4 relevant randomized controlled trials. Only 1 clinical trial of an SMS text messaging intervention for perioperative pediatric tonsillectomy has been published; it was conducted outside of North America [19] and had a high risk of bias [36]. Contributing our preliminary cohort study findings to this emerging academic literature can inform future trial designs for research teams facing similar pragmatic limitations and help to refine outcomes of interest to maximize translational research potential [37]. Second, TTTM is designed to support caregivers across the full perioperative period (ie, before, day of, and for 2 weeks after their child’s surgery) and was assessed using multiple measures (eg, analgesic use, caregiver self-efficacy, child pain levels, and health service use). Among technology-based pediatric-related intervention studies, most have measured only child and system outcomes [38] or measured them at only 1 postoperative time point [39,40]. Our comprehensive findings suggest that patient-level (eg, child pain) and system-level (eg, hospital visits) outcomes should be complemented with an assessment of the quality-of-care measures that help us to understand caregiver experiences (eg, caregiver distress) and behaviors across the perioperative period. Given the volume of tonsillectomy surgeries performed each year in North America [1], even modest individual-level improvements in pain management or improved perceptions of self-efficacy for managing care at home derived from brief SMS text messages could have significant real-world benefits. Finally, as caregivers’ role in pediatric perioperative care is vital [41], and they increasingly expect and prefer to receive information about surgical procedures through their smartphones [42], our study offers some of the earliest findings into how SMS text messaging as a modality might meet that need. Participants in our study, as well as other studies [43], report high satisfaction with health service–related SMS text messages, an even less intensive and complex technology than mobile apps. Caregivers actively engaged in learning about the skills and strategies offered through the texts. Given the large and potentially permanent migration to web-based supports and services during the COVID-19 pandemic, the need to support caregivers in using relevant technologies that can tailor what information they receive, when, and in what way may be even more pressing.

The early stage of research in this field presents numerous lines of future inquiry. Both groups in our study reported clinically significant levels of pain 3 days after surgery, and the embedded links to pain management strategies were engaged with the least. A better understanding of how SMS text messaging interventions might be optimized to improve adherence to best practice pain management strategies and promote the use of nonpharmacological pain management strategies could help to ensure that the most minimally invasive technology is used to produce optimal outcomes. Drawing from persuasive system design frameworks [44] and behavior change theories [45], there may be both content and functionality improvements that can be made to the intervention that might support improved pain management in particular. Second, monitoring and reporting on participant recruitment, satisfaction, feasibility, and outcome efficacy in demographically diverse populations will help to determine the utility and cultural relevance of these interventions. Our study, based in an east coast Canadian organization context, adds to the knowledge base but used a demographically homogenous sample. As concepts of pain, pain management [46], and caregiving [47] are deeply influenced by culture and ethnicity, it is critical, especially during this period of early evidence building, to expand our understanding of whether and how interventions such as TTTM should be tailored to be more culturally affirming [48].

### Limitations

Several study limitations should be noted. Our ability to conduct more robust analyses was limited because of sampling. Unforeseen delays occurred because of IT infrastructure approvals, and the COVID-19 pandemic limited the time frame for completing research activities. The use of historical control group data is prone to type I errors [21]; however, baseline demographic equivalence, no significant changes to the surgery itself, and the postoperative recommendations for parents between group conditions likely limited potential impacts. Given differences observed in recruitment and follow-up rates, some consideration of the external validity of the research is warranted; for example, changes to clinic and research staff may have introduced selection bias, and different recruitment and consent pathways (ie, the historical control group had a web-based consent option, whereas, for the intervention group, it was phone based) may confound the findings in ways we did not measure. It would be important for future research to be powered sufficiently to detect group differences and trial TTTM as a stand-alone intervention, not just as an adjunct to usual care. Data derived from this pilot study can be used to calculate the sample size for a future randomized controlled trial. The extent to which pandemic-related environmental factors for families (eg, caregivers spending more time at home with their children and children’s normal activities affected by public health restrictions) and health care organizations (eg, hospital visit requirements and physical distancing guidelines) affected the study results is unclear.

### Conclusions

Preliminary results from this prospective cohort intervention study with a historical control group revealed that TTTM had a positive impact on caregivers’ perioperative care experience. The results should be viewed with caution, given the unclear impacts of the COVID-19 pandemic on preoperative levels of caregiver distress, health service use, and typical caregiver-child interactions. Continued research into SMS text messaging interventions targeting pediatric perioperative experience is warranted, especially given caregivers’ high satisfaction with TTTM and high rates of texting in their everyday lives.

## Abbreviations

ED: emergency department
ENT: ear, nose, and throat
GHQ-12: General Health Questionnaire-12
OR: odds ratio
PPPM: Parents’ Postoperative Pain Measure
PSAM: Parenting Self-Agency Measure
REDCap: Research Electronic Data Capture
T1: time point 1
T2: time point 2
T3: time point 3
TTTM: *Tonsil-Text-To-Me*
